# Metal Ion-Based Supramolecular Self-Assembly for Cancer Theranostics

**DOI:** 10.3389/fchem.2022.870769

**Published:** 2022-05-20

**Authors:** Bing Chen, Chengchao Chu, En Ren, Huirong Lin, Yang Zhang, Peiyu Wang, Hong Yao, Ailin Liu, Gang Liu, Xinhua Lin

**Affiliations:** ^1^ Key Laboratory of Nanomedical Technology (Education Department of Fujian Province), School of Pharmacy, Nano Medical Technology Research Institute, Fujian Medical University, Fuzhou, China; ^2^ State Key Laboratory of Molecular Vaccinology and Molecular Diagnostics, Center for Molecular Imaging and Translational Medicine, School of Public Health, Xiamen University, Xiamen, China

**Keywords:** supramolecular therapeutic systems, metal-coordination-assisted, *in vivo* self-assembly, tumor microenvironment, personalized medicine

## Abstract

Metal-ion-based self-assembly supramolecular theranostics exhibit excellent performance in biomedical applications owing to their potential superiorities for simultaneous precise diagnosis, targeted drug delivery, and monitoring the response to therapy in real-time. Specially, the rational designed systems could achieve specific *in vivo* self-assembly through complexation or ionic interaction to improve tissue-specific accumulation, penetration, and cell internalization, thereby reducing toxicities of drugs in diagnostics and therapy. Furthermore, such imaging traceable nanosystems could provide real-timely information of drug accumulation and therapeutic effects in a non-invasive and safe manner. Herein, the article highlights the recent prominent applications based on the metal ions self-assembly in cancer treatment. This strategy may open up new research directions to develop novel drug delivery systems for cancer theranostics.

## Introduction

The usefulness of small-molecule therapeutic dyes has been widely studied in various therapeutic paradigms (e.g., phototherapy, sonodynamic therapy, and chemodynamic therapy); however, their efficacy is limited by instability, rapid clearance, and low tissue selectivity (Li et al., 2021). Supramolecular self-assembly which incorporate the functional moieties into supramolecular systems can improve their own performance such as prolonged half-life, enhanced stability and tissue-specific accumulation, is a promising strategy for a personalized therapeutic regimen (Wang et al., 2020; Chen Y. X et al., 2021). In addition, such supramolecular nanostructures can be used for multiple applications simultaneously, including diagnosis, drug delivery, and monitoring the spontaneous response to therapy. (Ren et al., 2017; Chen S et al., 2021). Based on these multiple advantages, an ever-expanding set of supramolecular architectures have been extensively explored, including block copolymers, metal-organic complexes (MOCs), and rationally designed peptides and so on (Dong et al., 2021). However, most of previously reported molecular self-assembly drug delivery systems have little availability in clinical practice, primarily due to the hurdles that can be associated with the biotoxic materials and biological side-effects. Supramolecular self-assembly based on therapeutic agents that are safe and close to clinical translation, which can avoid the potential safety issues and have significant potential for disease theranostics. Indeed, these theranostic systems have garnered considerable attention in multidisciplinary fields, and further progress toward their clinical application is expected in the near future. (Moore and Jokerst, 2019; Gao J et al., 2020).

Metal ions (e.g., iron, zinc, and calcium ions) play important roles in numerous physiological functions such as cellular homeostasis and enzymatic activities, and can also coordinate with functional molecules to spontaneously form an organized self-assembled supramolecular nanostructure with excellent biostability, biocompatibility, and safety (Qian and Xu, 2015). Iron, an essential element, is required for most living systems because it is the key constituent of fundamental cellular and organismal processes. Either too much or too little iron ions will cause serious health problems. For example, excess free iron accumulates in the liver when hepatic diseases (e.g., chronic hepatitis, hepatic fibrosis, and hepatocellular carcinoma) and hemochromatosis occur, which can cause oxidative damage to lipids, proteins, and DNA (Asare et al., 2006; Brissot et al., 2018; Wang and Babitt, 2019). Magnetic resonance imaging (MRI), a non-invasive imaging modality, has been widely employed for the clinical detection of the liver structure and functioning. However, the low specificity, iron quantification sensitivity, and the different iron forms (e.g., ferritin, hemosiderin, and labile iron) distinction inability limit the application of MRI in precision liver iron concentration detection (Wáng, 2021). Metal coordination-driven self-assembling photosensitizers in supramolecular systems can improve phototherapy efficacy and the safety of photosensitizers. The tumor environment-triggered coassembly strategy can overcome the critical issues of precise delivery and address the requirements to monitor therapeutic effects with non-invasive quantitative diagnosis (Chu et al., 2017). Ferric (Fe^3+^), zinc (Zn^2+^), and manganese (Mn^2+^) ions have been confirmed to interact with sulfonic acid and Lewis base groups at the terminal of Indocyanine green (ICG), a US FDA-approved theranostic dye for clinical use, to self-assemble into multi-level supramolecular systems (Shang et al., 2017; Chu et al., 2019; Zhang et al., 2021). Such metal-assisted multi-level self-assembly strategies offer significant advantages with respect to fabrication of hybrid imaging techniques that combine therapy for multimodal imaging-guided theranostics (Zhang et al., 2020; Du et al., 2021). For example, ICG interacting with an RGD peptide-modified Zn^II^-dipicolylamine-Arg-Gly-Asp (Bis(DPA-Zn)-RGD) complex showed more efficient ability to carry surviving small interfering RNA and to target pathological corneal tissues. Moreover, this ICG-linked complex, can self-assemble into a metal-organic nanostructures (MONs) to realize multimodal imaging-guided phototherapy and gene therapy, thus improving the corneal neovascularization synergistic therapy effect (Chu et al., 2020). Furthermore, *in situ* self-assembly of supramolecular systems can improve tissue-specific accumulation, multimodal imaging signals, and therapy efficacy, which possess good biocompatibility and stability. Additionally, the study of metal-ion-guided *in vivo* self-assembly is also helpful to understand the assembly characteristic of disease-related metal ions and the process of natural self-assembled supramolecular nanostructures in living systems (Guo et al., 2021).

**FIGURE 1:**
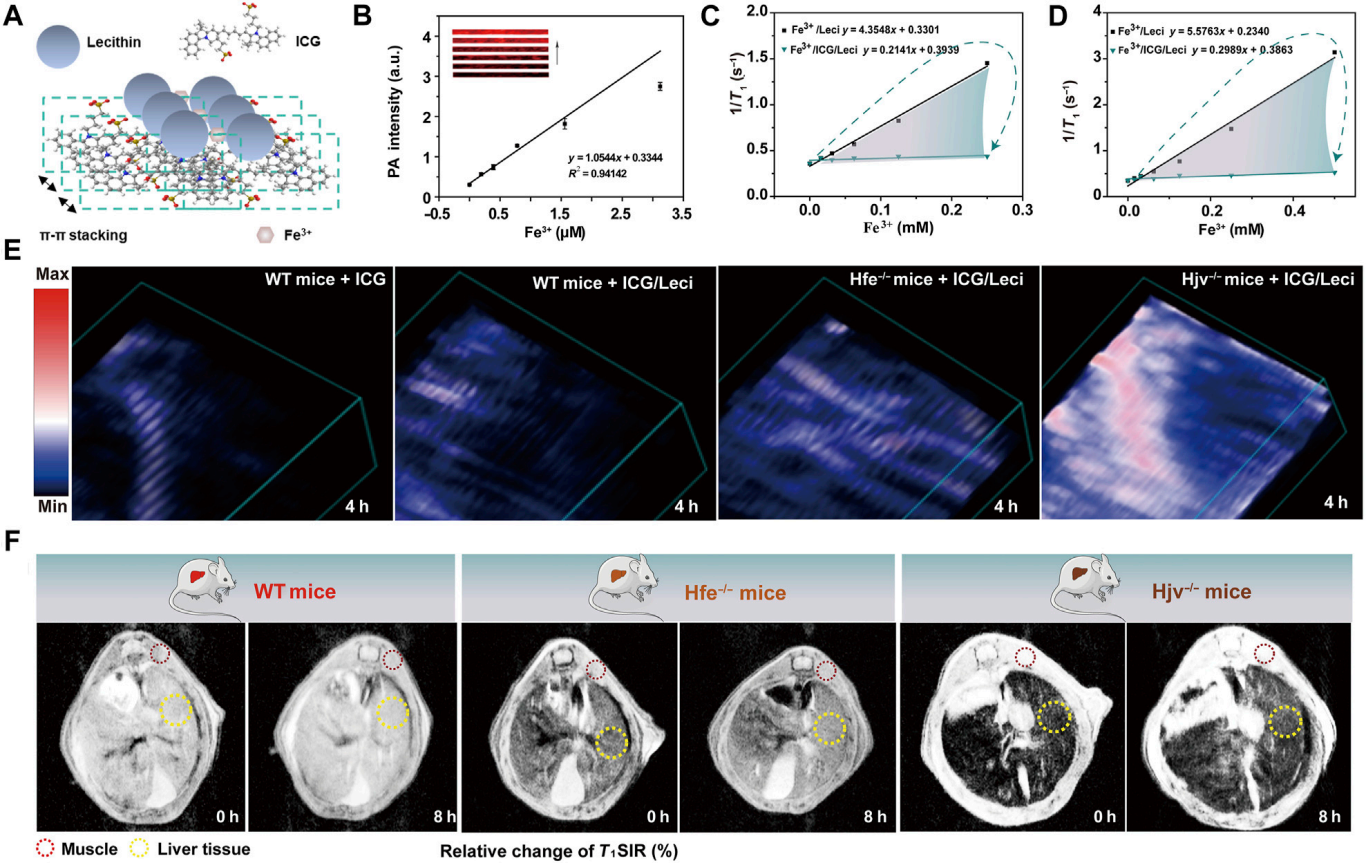
**(A)** Schematic illustration of Fe^3+^/ICG/Leci formation; **(B)** photoacoustic response of the probes at 890 nm to Fe^3+^ (0–3.12 μM) at pH 4.5; **(C)** r_1_ of Fe^3+^/Leci and Fe^3+^/ICG/Leci was measured in 1.5-T and **(D)** 9.4-T magnetic fields at pH 4.5; **(E)** representative photoacoustic images and **(F)** MRI images of mice models before and after intravenous injection of free ICG and ICG/Leci (ICG dose, 2.5 mg/kg) (Lin et al., 2021). Science Advances.

Lin et al. developed an *in situ* self-assembly ICG-based supramolecular system for simultaneous detection and treatment of hepatocellular carcinoma or other iron-overload disorders. Fe^3+^ was confirmed to coordinate with the sulfonic acid from ICG and form supramolecular nanoassemblies, which significantly decreased the single-MRI intensity and enabled rapid clearance from the body (approximately 50-fold lower than that of free Fe^3+^), thereby, addressing the clinical requirement for high-performance iron quantification while facilitating excess iron drainage (Figure 1A). The ICG-Lecithin (ICG/Leci) chelates with endogenous Fe^3+^ ions, which could more significantly reduce serum ferritin levels and increase iron excretion than free ICG and deferoxamine (DFO, a clinically prescribed iron depletion drug) (Lin et al., 2019). Meanwhile, ICG and ICG/Leci could significantly decrease the *T*_*1*_ signal intensity ratio (T_1_SIR) of Fe^3+^, producing a marked correlation between the *T*_*1*_SIR and the inner Fe^3+^ concentration, which could be repurposed as an MRI contrast and quantitative liver iron concentration measure (Lin et al., 2021). The metal-coordination-assisted *in situ* self-assembly supramolecular systems with the theranostic dye can promote iron ion-induced aggregation and further trigger unique optical properties (Ren et al., 2019). After incubation with Fe^3+^, the fluorescence intensity of ICG/Leci was reduced due to its coordination with Fe^3+^. As shown in Figures 1B–D, compared with ICG and DFO, ICG/leci showed that the optical absorption band appeared on a unique spectroscopic peak at 890 nm and obviously decreased from 550 to 880 nm, which resulted from the surface modification of ICG/Leci by free Fe^3+^. By taking advantage of photoacoustic imaging (PAI) of ICG/Leci-assembly of Fe^3+^ and MRI contrast changes (Figures 1E,F), which could provide valuable anatomical and functional information in a non-invasive manner, to observe iron depletion and quantitation, and comprehensively understand the therapeutic effects of *in vivo* self-assembly supramolecular on iron-overload disorders (Gao H et al., 2020). Furthermore, the ability of DFO, ICG, and ICG/Leci to promote iron excretion and elimination was evaluated in Hfe−/− mice, a model of hereditary hemochromatosis. The ICG/Leci supramolecular system exhibited significantly greater removal of excess iron *in vivo* without inducing renal injury. Based on the advantages of deep tissue diagnosis and real-time monitoring of therapeutic effects *in vivo*, the precise localization and multimodal imaging-guided therapy efficacy, the ICG/Leci supramolecular system is a promising theranostic agent for future research and clinical translation (Chen et al., 2020). Collectively, a supramolecular system was introduced to encapsulate Fe^3+^/ICG/leci through *in situ* self-assembly, which is a highly adaptable platform that integrates multimodal probes for fluorescence imaging, MRI, PAI, and synergetic therapy.

In addition to endogenous metal irons, exogenous essential mineral ions have also been explored for the design of a tissue environment-triggered supramolecular system with *in vivo* self-assembly. Chu et al. proposed a tumor stimuli-responsive co-assembly strategy to improve cancer therapy by *in situ* oxygen and generation of the novel sinoporphyrin sodium (DVDMS) nanotheranostic photosensitizer (nanoDVD). DVDMS is widely applied in phototherapy due to its ability to transform near-infrared (NIR) laser into heat and generate copious amounts of reactive oxygen species (ROS) (Ai et al., 2016). However, a few deficiencies still limit its application, such as non-selectively and low tumor penetrability. The MnO_2_ nanosheet has excellent efficiency for DVDMS loading, which can release out Mn^2+^, O_2_ and DVDMS under GSH and H_2_O_2_/H^+^ reduction in the tumor environment, to produce copious singlet state O_2_ (^1^O_2_) production, thus improving the phototherapy effect. Forthermore, as shown in Supplementary Figure S1 MnO_2_/DVDMS can self-assemble into nanoDVD *in vivo*, which can be monitored by activated photoacoustic/fluorescence/magnetic resonance imaging (Xuan et al., 2020; Yu et al., 2021). The tumor microenvironment-triggered supramolecular system has been demonstrated overall improved cancer therapy effect through the consumption of GSH, the production of O_2_ and ROS.

Furthermore, metal ion-guided supramolecular self-assembly nanoplatforms have multiple desirable functions, which can overcome the two major obstacles of drug delivery: the blood-brain barrier (BBB) and the blood–brain tumor barrier (Shi et al., 2017). Gao et al. developed an *in situ* assembled nanoplatform to achieve precise orthotopic multimodal imaging and imaging-guided thermal ablation through coordination-driven self-assembly supramolecular systems (Supplementary Figure S2A). The authors demonstrated that intelligent self-assemblies by upconversion nanocrystals could improve tumor-specific accumulation/retention for the phototherapy effect without damaging the skull and scalp in rodent models of orthotopic glioma (Supplementary Figures S2B,C). Specifically, Bis(DPA-Zn)-RGD nano-components exhibited good biocompatibility, improved neovascular-targeting, and excellent photothermal properties (Supplementary Figures S2B,C). Simultaneously, the tumor site of the photoacoustic signal increase in the glioma region of the mice was consistent with the determined using MRI (Supplementary Figures S2D,E). Moreover, nanoscale ICG-based nanoscale gold particles have been extensively applied in drug delivery and molecular imaging because of their tunable size, outstanding optical properties, and nontoxicity. Considering the ultra-small size ( ∼ 7 nm) of gold nanoparticles and the positive charge of Bis(DPA-Zn)-RGD with angiogenesis targeting, these materials can successfully cross the BBB to reach the tumor site, resulting in an unexpected therapeutic effect for orthotopic glioma.

**FIGURE 2:**
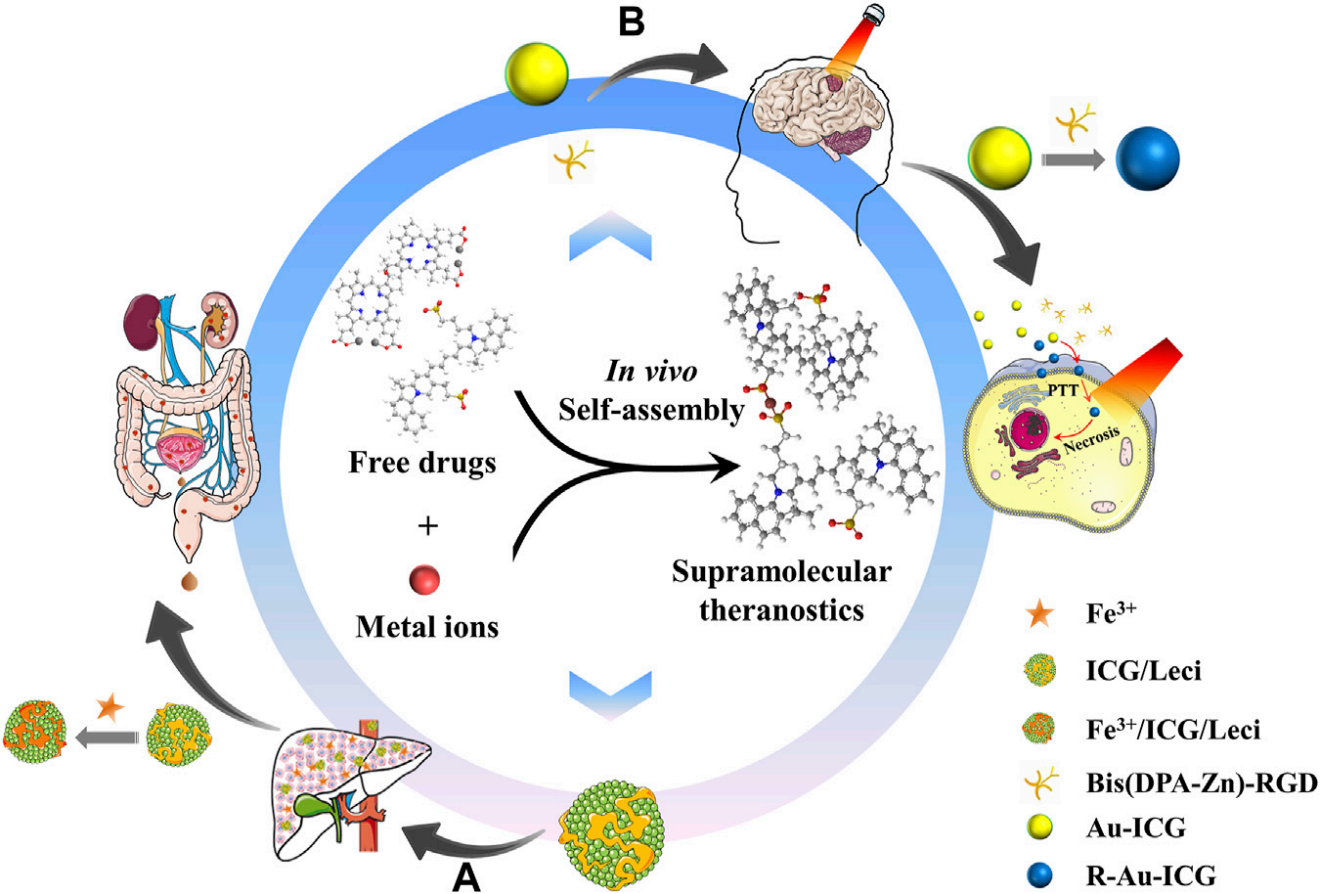
Schematic graph of metal-ion-guided *in vivo* self-assembly supramolecular for theranostic application. **(A)** Iron chelators of ICG/Leci will capture endogenous hepatic iron and self-assembled *in situ*, which can efficiently removing excess iron; **(B)** Positive charges and neovascular targeting properties of Bis(DPA-Ze)-RGD and ultrasmall particle size of Au-ICG could successfully cross the BBB and BBTB, and *in situ* assembled for imaging and therapy of orthotopic glioma.

In summary, rationally designed nanotheranostics that harness the metal-coordination-assisted molecular assembly of therapeutic agents can simultaneously diagnose, deliver drugs, and monitor the response to therapy in real-time. The high specificity and sensitivity of theranostic dye-bearing supramolecular nanoplatforms integrate multimodal imaging functions as well as synergetic therapy, which demonstrates significant potential for clinical early-stage diagnosis and effective treatment (Figure 2). Compared with traditional methods for preparing an ICG-based supramolecular therapeutic, metal ion-guided self-assembly supramolecular systems have advantages such as improved drug loading and tissue-specific targeting capacity, resulting in precise orthotopic multimodal imaging-guided drug delivery. It is believed that this carrier-free delivery system, which exhibits improved biocompatibility and outstanding performance in terms of biostability and non-invasive molecular imaging, can be applied from beach to bedsides. However, the comprehensive pharmacokinetic behavior and safety evaluation of metal-organic nanoparticles are needed before the clinical application.

## Data Availability

The original contributions presented in the study are included in the article/Supplementary Material, further inquiries can be directed to the corresponding authors.

## Abbreviations

BBB, Blood-brain barrier; BBTB, Blood-brain tumor barrier; DVDMS, Sinoporphyrin sodium; ICG, Indocyanine green; MOCs, Metal-organic complexes; PAI, Photoacoustic imaging; ROS, Reactive oxygen species; T_1_SIR, *T*_*1*_ signal intensity ratio.
