# Supplementary material for: Metal Ion-Based Supramolecular Self-Assembly for Cancer Theranostics
Source: Front Chem. 2022 May 20;10:870769. doi: 10.3389/fchem.2022.870769 (PMC9163678; doi:10.3389/fchem.2022.870769)
Supplement: Supplementary file 1 [file DataSheet1.docx]

**Supplementary Material**

**Metal Ion-based Supramolecular Self-assembly for Cancer Theranostics**

Bing Chen^1,2†^, Chengchao Chu^2†^, En Ren^2^, Huirong Lin^2^, Yang Zhang^2^, Peiyu Wang^2^, Hong Yao^1^, Ailin Liu^1^, Gang Liu^2*^, and Xinhua Lin^1*^

^1^Key Laboratory of Nanomedical Technology (Education Department of Fujian Province), School of Pharmacy, Nano Medical Technology Research Institute, Fujian Medical University, Fuzhou, Fujian 350122, P.R. China

^2^State Key Laboratory of Molecular Vaccinology and Molecular Diagnostics & Center for Molecular Imaging and Translational Medicine, School of Public Health, Xiamen University, Xiamen, Fujian 361102, P.R. China

*** Correspondence:**

Prof. Xinhua Lin, School of Pharmacy, Nano Medical Technology Research Institute, Fujian Medical University, Fuzhou, Fujian 350122, P.R. China.

E-mail: 13906909638@163.com;

Prof. Gang Liu, State Key Laboratory of Molecular Vaccinology and Molecular Diagnostics & Center for Molecular Imaging and Translational Medicine, School of Public Health, Xiamen University, Xiamen, Fujian 361102, P.R. China.

E-mail: Gangliu.cmitm@xmu.edu.cn.

^†^These authors have contributed equally to this work and share first authorship

**List of Supplementary Material Captions**

**Figure. S1** (A) Schematic illustration of Mn2+ ionguided in vivo self-assembly with DVDMS for photothermal/photodynamic therapy; (B) T1-weighted MR images, (C) thermal images, (D) PA images, and (E) fluorescence images before and after injection of MnO2/DVDMS (Chu et al., 2017). Advanced Materials.

**Figure. S2** (A) Schematic illustration of the process across the BBB and BBTB of Bis(DPA-Ze)-RGD and Au-ICG; (B) near-IR imaging of mice bearing orthotopic glioma at various time points; (C) fluorescence imaging of tumors from mice bearing glioma treated with R/Au-ICG nanoclusters ex vivo; (D) photoacoustic detection of R/Au-ICG nanoclusters and (E) MRI signal change in orthotopic glioma mice (Gao et al., 2020a). ACS Applied materials & interfaces.


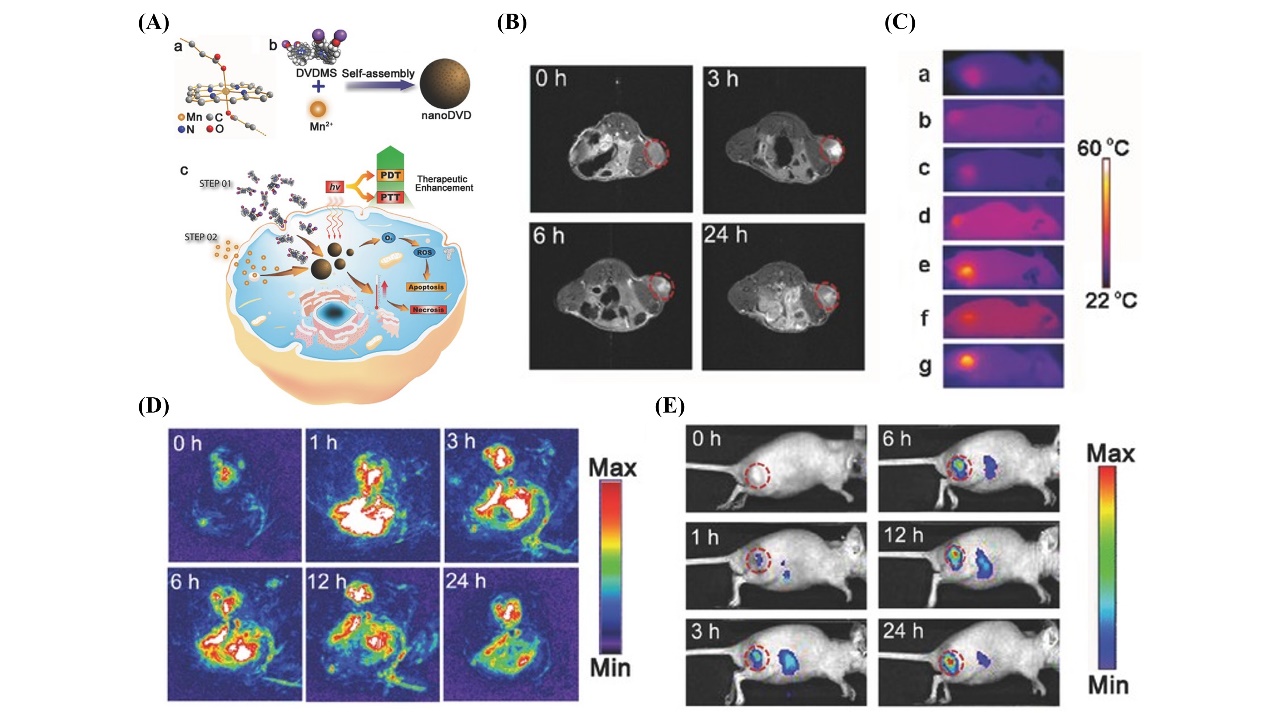


**Figure. S1** (A) Schematic illustration of Mn2+ ionguided in vivo self-assembly with DVDMS for photothermal/photodynamic therapy; (B) T1-weighted MR images, (C) thermal images, (D) PA images, and (E) fluorescence images before and after injection of MnO2/DVDMS (Chu et al., 2017). Advanced Materials.


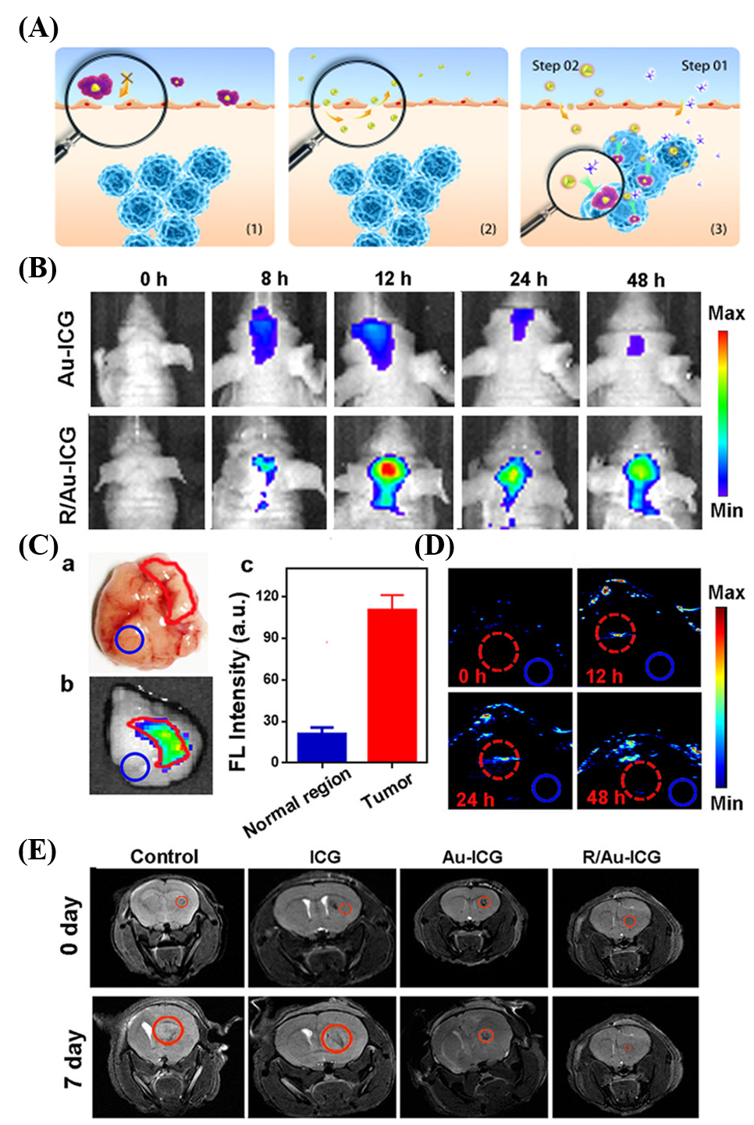


**Figure. S2** (A) Schematic illustration of the process across the BBB and BBTB of Bis(DPA-Ze)-RGD and Au-ICG; (B) near-IR imaging of mice bearing orthotopic glioma at various time points; (C) fluorescence imaging of tumors from mice bearing glioma treated with R/Au-ICG nanoclusters ex vivo; (D) photoacoustic detection of R/Au-ICG nanoclusters and (E) MRI signal change in orthotopic glioma mice (Gao et al., 2020a). ACS Applied materials & interfaces.
